# Interaction of Citrinin with Human Serum Albumin

**DOI:** 10.3390/toxins7124871

**Published:** 2015-12-01

**Authors:** Miklós Poór, Beáta Lemli, Mónika Bálint, Csaba Hetényi, Nikolett Sali, Tamás Kőszegi, Sándor Kunsági-Máté

**Affiliations:** 1 Department of Pharmacology and Pharmacotherapy, Toxicology Section, University of Pécs, Szigeti út 12, Pécs H-7624, Hungary; 2 Department of General and Physical Chemistry, University of Pécs, Ifjúság útja 6, Pécs H-7624, Hungary; blemli@gamma.ttk.pte.hu (B.L.); kunsagi@gamma.ttk.pte.hu (S.K.-M.); 3 János Szentágothai Research Center, Ifjúság útja 20, Pécs H-7624, Hungary; niki26@gmail.hu (N.S.); koszegitam@gmail.com (T.K.); 4 Department of Biochemistry, Eötvös Loránd University, Pázmány sétány 1/C, Budapest 1117, Hungary; monibalint18@gmail.com; 5 MTA-ELTE Molecular Biophysics Research Group, Hungarian Academy of Sciences, Pázmány sétány 1/C, Budapest 1117, Hungary; csabahete@yahoo.com; 6 Department of Laboratory Medicine, University of Pécs, Ifjúság útja 13, Pécs H-7624, Hungary

**Keywords:** citrinin, human serum albumin, fluorescence spectroscopy, ultrafiltration, species differences

## Abstract

Citrinin (CIT) is a mycotoxin produced by several *Aspergillus*, *Penicillium*, and *Monascus* species. CIT occurs worldwide in different foods and drinks and causes health problems for humans and animals. Human serum albumin (HSA) is the most abundant plasma protein in human circulation. Albumin forms stable complexes with many drugs and xenobiotics; therefore, HSA commonly plays important role in the pharmacokinetics or toxicokinetics of numerous compounds. However, the interaction of CIT with HSA is poorly characterized yet. In this study, the complex formation of CIT with HSA was investigated using fluorescence spectroscopy and ultrafiltration techniques. For the deeper understanding of the interaction, thermodynamic, and molecular modeling studies were performed as well. Our results suggest that CIT forms stable complex with HSA (log*K* ~ 5.3) and its primary binding site is located in subdomain IIA (Sudlow’s Site I). *In vitro* cell experiments also recommend that CIT-HSA interaction may have biological relevance. Finally, the complex formations of CIT with bovine, porcine, and rat serum albumin were investigated, in order to test the potential species differences of CIT-albumin interactions.

## 1. Introduction

Mycotoxins are toxic secondary metabolites produced naturally by filamentous fungi. Due to their wide occurrence, mycotoxin contamination of various foods, drinks, and animal feed is unavoidable, causing serious health problems for humans and animals [1]. The nephrotoxic mycotoxin citrinin (CIT) is produced by *Aspergillus*, *Penicillium*, and *Monascus* fungi and appears mainly in various cereals (e.g., maize, rye, oat, barley, wheat, and rice); however, CIT occurs in other foodstuffs, as well (e.g., pomaceous fruits, black olive, spices, cheese, *etc.*) [2,3]. The wide occurrence of CIT is associated with a relatively high thermal stability making its removal from contaminated sources more difficult [4]. Recent studies suggest apoptosis induction and cell cycle arrest as its main toxic impacts; however, the exact mechanism of action of CIT is not clearly understood yet [5,6,7]. Relatively little information is available regarding the toxicokinetics of CIT. Some studies suggest that CIT is taken up by kidney cells through organic anion transporters [8,9]. Furthermore, recent investigations highlighted that both CIT and its inactive metabolite dihydrocitrinone [10] are present in measurable levels in human blood and urine [11,12].

Human serum albumin (HSA) is the most abundant plasma protein in human circulation. In addition to its role in the maintenance of oncotic pressure and pH of the blood, it also has antioxidant and pseudo-enzymatic activities [13]. In addition, one of the most important functions of HSA is the binding (and in this route the transport) of numerous endogenous molecules, drugs, and xenobiotics; therefore, HSA can play a major role in their pharmacokinetics/toxicokinetics in many cases [13,14]. Very relevant interactions of HSA with mycotoxins occur, e.g., in the case of ochratoxin A which shows structural similarity with citrinin [15,16]. However, previous studies demonstrated the presence of interaction between CIT and some albumin species [17,18], the complex formation of CIT with human serum albumin is poorly characterized.

In this study the interaction of CIT with human serum albumin (HSA) was investigated using fluorescence spectroscopy, ultrafiltration, and molecular docking studies. Our main goals were to determine the stability of CIT-HSA complex, as well as to identify the primary binding site of CIT on the HSA molecule. For deeper understanding of CIT-HSA complex formation, thermodynamic studies were performed, as well. Furthermore, CIT-albumin interaction was tested on kidney cells applying an *in vitro* cell culture, in order to examine its potential relevance. Finally, interactions of CIT with bovine, porcine, and rat serum albumin were also investigated to explore the possible species differences regarding CIT-albumin complexes. Our results clearly demonstrate that CIT forms a stable complex with albumin suggesting the potential *in vivo* relevance of the interaction, which one should keep in mind in the future.

## 2. Results and Discussion

### 2.1. Binding Constant of Citrinin-HSA Complex

**Figure 1:**
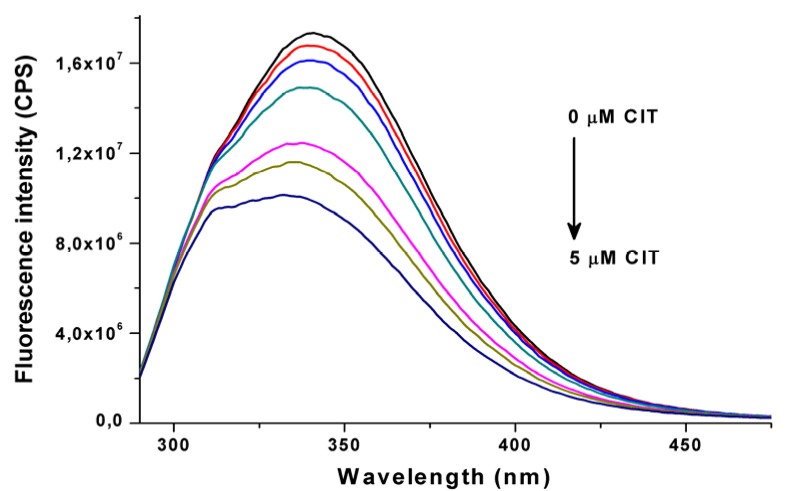
Fluorescence emission spectra of human serum albumin (2 µM) in the presence of increasing CIT concentrations (0, 0.25, 0.5, 1, 2, 3 and 5 µM) in PBS (pH 7.4) (λ_exc_ = 280 nm).

Citrinin shows fluorescence properties only under an acidic environment (below pH 5) [19]; therefore, fluorescence quenching of HSA by CIT was examined in order to investigate CIT-HSA interaction at physiological pH (pH 7.4; λ_exc_ = 280 nm, λ_em_ = 340 nm). Fluorescence emission spectra of HSA (2 μM) in the absence and in the presence of increasing CIT concentrations (0.25–5.0 μM) are plotted in Figure 1. Even the presence of CIT in nanomolar concentrations resulted in the significant fluorescence quenching of HSA. During these measurements 5.32 ± 0.01 was determined as log*K* value at 25 °C (see details in Section 3.2.), suggesting a stable and relevant interaction between CIT and HSA. The determined binding constant is similar to that of described regarding warfarin-HSA interaction (log*K* ~ 5.3), which results in approximately 99% bonding in plasma and, therefore, the long half-life of warfarin in human circulation [15,20]. Due to the blue shift of the fluorescence emission maximum of HSA in the presence of CIT, determination of a binding constant was repeated using different emission wavelengths (330–350 nm). No significant differences were observed compared to the stability constant determined at a wavelength of 340 nm. Our calculations strongly suggest 1:1 stoichiometry of the CIT-HSA complex, indicating the presence of one high-affinity binding site of CIT on the HSA molecule.

The impact of pH on CIT-HSA interaction was also tested. Since albumin presents in its N (neutral) form between pH 4.3–8.0 [13], during our experiments CIT-HSA complex formation was investigated at pH 6.0, 7.0 and 7.8 in PBS buffer (T = 25 °C). Under these circumstances, only slight changes of the stability of CIT-HSA complex was observed (pH 6.0 → log*K* = 5.25 ± 0.01; pH 7.0 → log*K* = 5.28 ± 0.01; pH 7.8 → log*K* = 5.12 ± 0.01). However, at pH 7.8 a larger decrease of the log*K* value was noticed compared to log*K* values determined at pH 6.0 and 7.0.

### 2.2. Ultrafiltration Experiments

**Figure 2:**
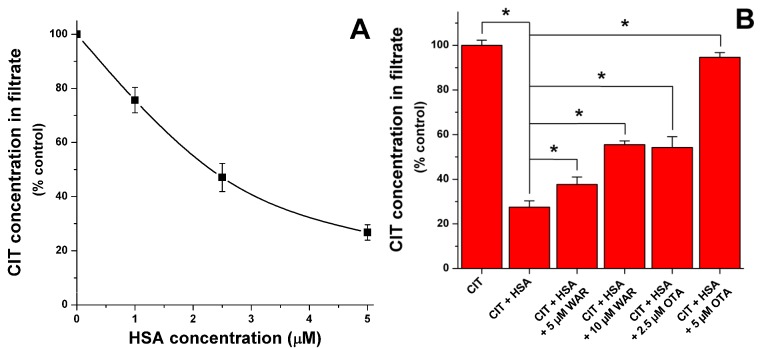
(**A**) Citrinin levels in ultrafiltrates (1 μM CIT) in absence and presence of increasing HSA concentrations; and (**B**) influence of warfarin and ochratoxin A (in the presence of 1 μM CIT + 5 μM HSA) on citrinin concentrations in filtrate (see further details in Section 3.4.) (* *p* < 0.05).

To confirm the presence of CIT-HSA interaction, as well as to identify the primary binding site of CIT on HSA molecule, ultrafiltration experiments were performed. Since ultrafiltration is a relatively long process (10 min in our experiments), this technique is not suitable to quantify the binding constant. However, ultrafiltration is highly suitable to clearly demonstrate the complex formation and to investigate different competitive interactions (and in this way to identify the binding site with specific site markers) [21,22]. As Figure 2A demonstrates, the presence of increasing HSA concentrations (1, 2.5, and 5 μM) led to the significant decrease of CIT in the filtrate. Since HSA is an approximately 67 kDa sized macromolecule [15], it is not able to pass through the filter unit with a 10 kDa molecular weight cut-off value. For this reason only the free (not albumin-bound) molecules will reach the filtrate. These results confirm the presence of a relevant interaction between CIT and HSA.

Thereafter, the influence of site markers on CIT-HSA interaction was tested. Warfarin and ibuprofen are commonly applied site markers of Sudlow’s site I and II, respectively [13]. HSA contains three domains (I, II, and III) and each domain is built up from two subdomains (A and B). A large cavity in subdomain IIIA hosts Sudlow’s site II (or drug binding site II); the typical ligands of this binding site are the non-steroidal anti-inflammatory drug ibuprofen and benzodiazepines (e.g., diazepam), and Sudlow’s site II is preferred by several aromatic carboxylates, as well [13]. Sudlow’s site I (or drug binding site I) is located in a cavity in subdomain IIA; however, it is smaller compared to the cavity in Sudlow’s site II. The ligands of Sudlow’s site I are mainly bulky heterocyclic anions and its typical ligand is warfarin. Tyr150 amino acid presumably plays a major role regarding drug-HSA interactions, because its hydroxyl group commonly forms hydrogen bond during the complex formation of HSA with different ligands [13]. Before ultrafiltration, samples contained 1 μM CIT and 5 μM HSA in absence and presence of warfarin or ibuprofen (5 or 10 μM). In presence of ibuprofen no significant changes of CIT concentrations were observed in the filtrate (data not shown). In contrast, the presence of warfarin caused considerable increase of CIT in the filtrate suggesting the significant displacement of CIT by warfarin (Figure 2B). It was plausible to hypothesize that CIT is a Sudlow’s site I ligand similarly to the other mycotoxin ochratoxin A (OTA) which shows structural similarity with CIT [23]. Furthermore, the molecular location of the binding site of OTA and warfarin is almost completely the same [24]. For this reason, molecular displacement of CIT by OTA was also investigated. As Figure 2B demonstrates, the presence of OTA resulted in the significant elevation of CIT in the filtrate, showing remarkable displacement of CIT even by 2.5 and 5 μM OTA. It is not surprising because OTA binds to HSA with much higher affinity (log*K* = 7.4–7.6 at 25 °C) compared to warfarin (and to citrinin as well) [15,20,25,26]. Since equimolar amount of OTA is able to bind most of the HSA molecules [26] and because of the much higher affinity of OTA toward HSA, almost complete displacement of CIT (1 μM) was observed in the presence of 5 μM OTA.

The strong quenching of the fluorescence of HSA by CIT as well as the remarkable displacement of CIT from HSA in presence of Sudlow’s site I ligands warfarin and ochratoxin A show a circumstantial evidence that the primary binding site of CIT is located in Sudlow’s site I (subdomain IIA).

### 2.3. Fluorescence Investigation of Molecular Displacement of Warfarin and Ochratoxin A from HSA by Citrinin

To support further the binding site of CIT obtained and the quantified stability constant of CIT-HSA complex, competitive interaction of CIT with warfarin and ochratoxin A was investigated using our models published earlier [20,25,26].

**Figure 3:**
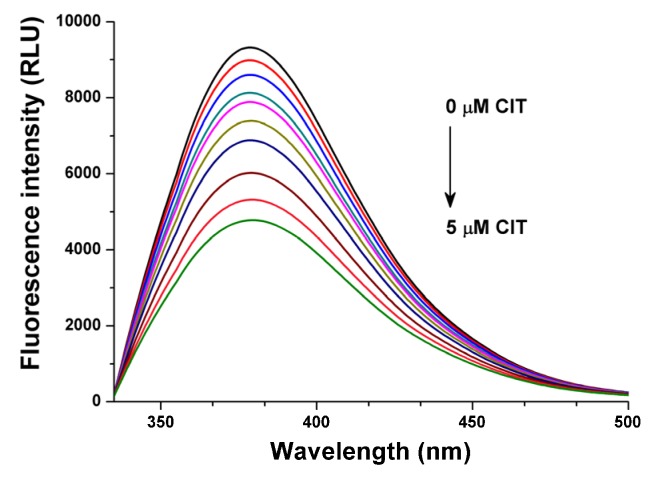
Fluorescence emission spectra of warfarin (1 μM) in the presence of HSA (3.5 μM) and increasing CIT concentrations (0, 0.25, 0.5, 0.75, 1, 1.5, 2, 3, 4 and 5 μM) in PBS (λ_exc_ = 317 nm).

Warfarin shows fluorescence excitation and emission maxima at 309 nm and 389 nm wavelengths, respectively. However, in presence of HSA warfarin exerts more than 15-fold higher fluorescence signal, while the excitation maximum of HSA-bound warfarin is shifted to 317 nm and providing the maximum fluorescence emission intensity at 379 nm [20]. In the presence of increasing CIT concentrations (0.25–5.0 μM) significant decrease of the fluorescence intensities of warfarin was observed (Figure 3), indicating the molecular displacement of warfarin from the surface of HSA molecule [20].

**Figure 4:**
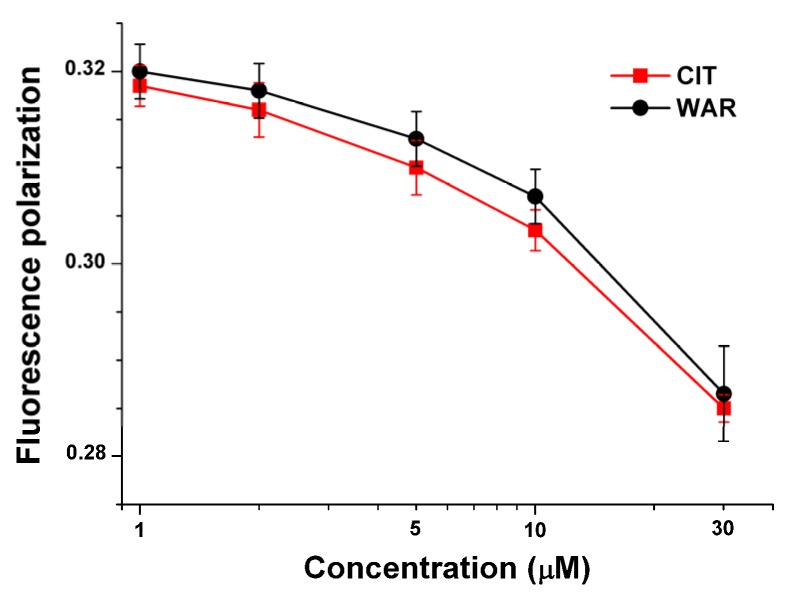
Fluorescence polarization data of OTA (1 μM) in the presence of HSA (1.4 μM) and increasing amounts of citrinin or warfarin in PBS (λ_exc_ = 393 nm, λ_em_ = 446 nm).

As it was discussed previously (in Section 2.2.), ochratoxin A binds to HSA with very high affinity. Since OTA exerts strong fluorescence signal, the complex formation of OTA with HSA can be easily followed by fluorescence polarization technique [23,25,26]. Previous studies also highlighted that fluorescence polarization-based models are able to describe the molecular displacement of OTA by different drug molecules or other compounds [25,26]. Since the calculated binding constant of CIT-HSA complex was very similar compared to warfarin-HSA complex, fluorescence polarization (P) values of OTA (1 μM) in presence of HSA (1.4 μM) were determined in the absence and presence of CIT or warfarin (1–30 μM). Figure 4 shows that both CIT and warfarin result in the decrease of fluorescence polarization of OTA in a concentration dependent fashion. Since the rotational freedom of free (not protein-bound) OTA is much higher (therefore its P value is substantially lower: *P*_OTA_ = 0.010–0.015, *P*_OTA-HSA_ = 0.320–0.330) compared to its albumin-bound form, the decrease of fluorescence polarization values suggests the molecular displacement of OTA from HSA [25,26]. Furthermore, as it was demonstrated in previous studies, depending on the binding affinity of the competitor molecule the displacing ability could be higher or lower [25,26]. The very similar P values in presence of CIT and warfarin recommend that the displacing abilities of CIT and warfarin and therefore the binding constants of CIT-HSA and warfarin-HSA complexes are nearly the same; which is in a very good agreement with the results of fluorescence quenching experiments (see in Section 2.1.).

Based on steady-state fluorescence spectroscopic and fluorescence polarization studies, CIT is able to displace both warfarin and ochratoxin A form HSA. These results confirm further that CIT is a Sudlow’s site I ligand, strongly supporting our observations regarding the ultrafiltration experiments (see in Section 2.2.).

### 2.4. Thermodynamic Studies

In order to investigate the temperature dependence of CIT-HSA interaction, binding constants were determined (using Equation (1)) between 25 and 40 °C. The measured log*K* values of CIT-HSA complex at different temperatures are summarized in Table 1. Data show higher stability of the complexes at lower temperatures reflecting ground state complexes. Thermodynamic parameters were also determined (using Equation (2)) in order to get deeper insight regarding the nature of the binding forces. These interaction forces are usually van der Waals interactions, hydrophobic forces, multiple hydrogen bonds or electrostatic interactions. The calculated negative *∆G* value (−29.96 kJ·mol^−1^) suggests the spontaneous binding process between CIT and HSA; it is within the typical range of noncovalent interactions. *∆H* and *∆S* values of CIT-HSA complex formation were −24.15 kJ·mol^−1^ and +20.90 J·mol^−1^·K^−1^, respectively. These thermodynamic parameters are mostly analyzed from the point of view of Ross and Subramanian [27]. During the interactions between proteins and other molecules, the dominant role of van der Waals forces and hydrogen bond formation typically results in negative values of both enthalpy and entropy changes. From the point of view of the solvent molecules, positive value of the entropy change indicates hydrophobic interaction between the macromolecule and the ligand, supposing the decomposition of the solvation shells of the interacting molecules (or a part of them) leading to less ordered structure of water molecules. Furthermore, the negative enthalpy change in combination with the positive entropy change suggest the presence of specific electrostatic interaction. According to the calculated *∆H* and *∆S* values the interaction seems to be an enthalpy driven process therefore electrostatic forces may play a major role during the CIT-HSA complex formation.

**Table 1:** Temperature dependence of citrinin-HSA interaction compared to warfarin-HSA complex formation (log*K* values of warfarin-HSA complex are derived from the study of Oester *et al.* [28]).

**T** (°C)	25	30	35	40
**log*K*** (±SD) (CIT-HSA)	5.32 ± 0.01	5.26 ± 0.01	5.19 ± 0.01	5.11 ± 0.01
**T** (°C)	25	-	37	42
**log*K*** (warfarin-HSA)	5.38	-	5.31	5.28

### 2.5. Molecular Modeling Studies

**Figure 5:**
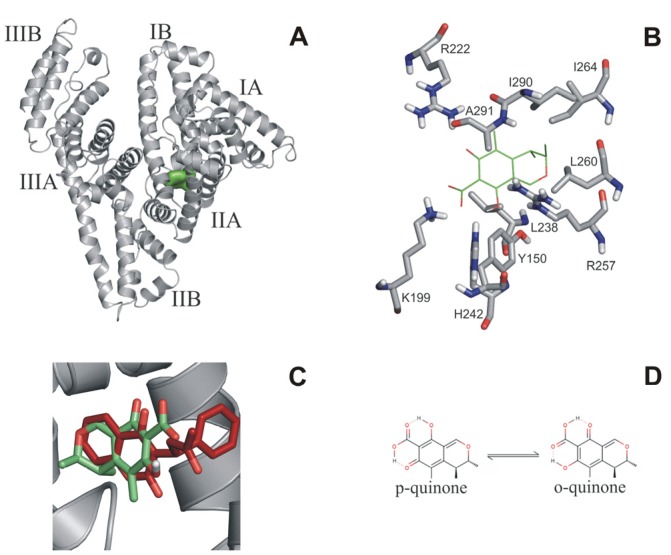
(**A**) Docked conformation of citrinin (green surface) in Sudlow’s site I on HSA (grey cartoon); (**B**) Docked p-quinone is represented as green sticks and the interacting amino acids in grey; (**C**) Match between the bound conformations of warfarin (red sticks) and citrinin (green sticks) in Sudlow’s site I. The binding conformation of warfarin was extracted from a previously determined warfarin-HSA complex structure (PDB code 1h9z) after superimposition of the HSA parts; (**D**) Lewis structure of tautomeric forms of citrinin.

A blind docking search was performed for the binding site(s) of citrinin on the entire surface of HSA. The search resulted in nine and ten representative binding positions for the o- and p-quinone forms of citrinin, respectively. For both forms, the best docked binding position with the lowest calculated *ΔG* found the well-known Sudlow’s site I [13,29] located in the cavity of subdomain IIA that is composed by all six helices of the subdomain and a loop-helix feature (residues 148–154) from IB (Figure 5A). Analysis of the contacts between the best docked citrinin and HSA (Figure 5B) showed that both o- and p-quinone forms had H-bonds with R222, H242 and R257. Salt bridges were observed for both o-quinone (K199) and p-quinone (K199, H242) forms. Hydrophobic contacts also play an important role in binding of citrinin with L238, I264, I290 (o-quinone), and L260, I264, and A291 (p-quinone form) residues involved. The above mentioned interacting residues are known as part of Sudlow’s site I (Figure 5A,B), and also as the binding site of warfarin (Figure 5A). For a direct comparison, the binding conformation of warfarin [30] was extracted from a previously determined warfarin-HSA complex structure (PDB code 1h9z) after superimposition of the HSA parts. A very good match was found between the bound conformations of warfarin and citrinin (Figure 5C). Thus, blind docking identified Sudlow’s site I as a primary binding position of citrinin on the surface of HSA. Thermodynamic measurements also suggest an enthalpic binding process which is based on the interactions between electron rich citrinin and the positively charged residues (K195, K199, R218 and R222) of Sudows’s site I. A good agreement between the measured *∆G* of CIT (−30.0 kJ·mol^−1^) and the calculated *∆G* value of the p-quinone form of CIT (−31.5 kJ·mol^−1^, Figure 5B,D) suggest that this tautomer is the most important in HSA binding. Previous studies also suggest that the p-quinone form may be the primary unbound tautomer (60%) in solution, as well [31].

### 2.6. Influence of Albumin on the Citrinin-Induced Toxicity *in Vitro*

**Figure 6:**
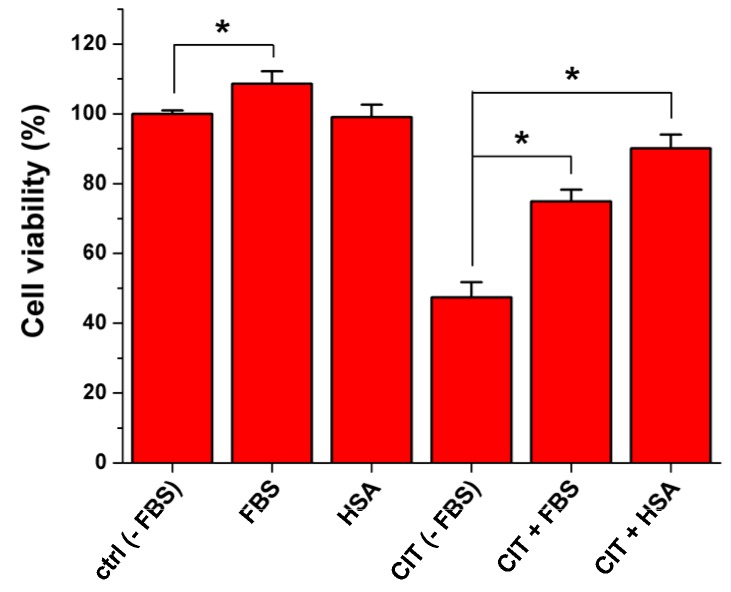
Influence of FBS (10%) and HSA (40 g/L) on viability of MDCK cells in the absence and in the presence of CIT (100 μM) after 24-h treatment (* *p* < 0.05).

In order to investigate the influence of albumin on the toxic impact of CIT, MDCK cells were treated with CIT in the absence and in the presence of FBS (fetal bovine serum) or HSA. To quantify the required CIT concentration which leads to serious viability loss, MDCK cells were exposed to increasing amounts of CIT (0–100 μM) in the absence of FBS for 24-h. Treatment of MDCK cells with 100 μM CIT caused approximately 50% decrease of cell viability (Figure S1); therefore, during the following experiments this concentration was applied. Presence of HSA (40 g/L) or FBS (10%) alone caused no or slight (but significant) changes of cell viability, respectively (compared to that of control); however, CIT-induced viability loss was considerably alleviated by both FBS and HSA (Figure 6). Aside from bovine serum albumin (BSA), FBS contains several further constituents (e.g., hormones and growth factors), and some of these factors enhance cell growth and cell division. It explains the positive effect of FBS even in the absence of CIT. Furthermore, the formation of stable complexes of different ligands with albumin results in the slower (and presumably incomplete) cellular uptake of the ligand molecules. Due to its toxic nature, CIT has negative effects on cell viability; therefore, the potential inhibition of its cellular uptake is beneficial. The CIT-induced viability loss was spectacularly alleviated by co-treatment of toxin-exposed cells with FBS (containing BSA) or HSA. This observation strongly suggests that the complex formation of CIT with albumin resulted in the decreased cellular uptake of CIT by MDCK cells. Furthermore, our results highlight that the presence of FBS in cell medium is important during *in vitro* experiments if one would like to mimic *in vivo* conditions.

### 2.7. Interaction of Citrinin with Bovine, Porcine, and Rat Serum Albumin

To test the potential species differences regarding CIT-albumin interactions, complex formations of CIT with bovine (BSA), porcine (PSA), and rat serum albumin (RSA) were also investigated. Despite of the structural differences of albumins (there is an approximately 25%–30% variability even in their amino acid sequences), their biological behavior (including their ligand binding properties) is usually very similar [15]. However, sometimes major differences occur: e.g., the complex stability of the mycotoxin ochratoxin A with HSA is 15-fold and 30-fold higher compared to its complexes with BSA and RSA, respectively [15]. Binding constants of CIT with albumins were determined with fluorescence quenching method under the same conditions than in Section 2.1. (using Equation (1)). Even if the binding constants of the four tested albumins with CIT show some differences (Table 2), we did not observe dramatic discrepancies, like in the case of ochratoxin A. The most stable complex of CIT is formed with RSA, followed by HSA, BSA, and PSA. These data suggest that albumin binding characteristics of CIT is similar in different species indicating that the albumin binding properties of CIT in humans may be modeled well by animal studies.

**Table 2:** Binding constants of CIT-albumin complexes in PBS (pH 7.4 at 25 °C).

Tested albumins	HSA	BSA	PSA	RSA
**log*K*** (±SD)	5.32 ± 0.01	5.05 ± 0.05	4.96 ± 0.05	5.50 ± 0.02

## 3. Experimental Section

### 3.1. Reagents

Citrinin (CIT), human serum albumin (HSA), bovine serum albumin (BSA), porcine serum albumin (PSA), rat serum albumin (RSA), warfarin (WAR), ochratoxin A (OTA), ibuprofen, DMEM (Dulbecco’s Modified Eagle’s Medium)—high glucose (4500 mg/L), fetal bovine serum (FBS), penicillin/streptomycin solution (all from Sigma-Aldrich, Saint Louis, MO, USA), calcein acetoxymethyl ester (CAM; from Thermo Fisher Scientific, Waltham, MA, USA) were used as received. All other reagents were of analytical or spectroscopic grade. 5000 μM stock solution of CIT was prepared in spectroscopic-grade ethanol (Reanal) and stored at −20 °C. In order to mimic extracellular physiological conditions, CIT-albumin interactions were investigated in phosphate-buffered saline (PBS, pH 7.4).

### 3.2. Fluorescence Spectroscopic Measurements

A Fluorolog τ3 spectrofluorometric system (Jobin-Yvon/SPEX) and Hitachi F-4500 fluorescence spectrophotometer were used for steady-state fluorescence and fluorescence polarization measurements. All analyses were performed at +25 °C except thermodynamic studies.

Binding constants (*K*) were determined using fluorescence quenching method, where increasing CIT concentrations (0–5 μM) were added to standard amount of albumin (2 μM). Fluorescence emission spectra were recorded in PBS buffer (pH 7.4; λ_exc_ = 280 nm). Similarly to our previous studies [15,16], *K* values of CIT-albumin complexes were quantified by non-linear fitting with Hyperquad2006 program package (Protonic Software) assuming 1:1 stoichiometry:

(1) $$I=I_{0}+\frac{I_{AC}-I_{0}}{2{\left[A\right]}_{0}}\left({\left[A\right]}_{0}+{\left[C\right]}_{0}+\frac{1}{K}-\sqrt{{\left({\left[A\right]}_{0}+{\left[C\right]}_{0}+\frac{1}{K}\right)}^{2}-4{\left[A\right]}_{0}{\left[C\right]}_{0}}\right)$$

where *I* denotes the fluorescence emission intensity of albumin in presence of CIT; *I*_0_ denotes the fluorescence emission intensity of albumin in absence of CIT; *I_AC_* denotes the fluorescence emission intensity of pure albumin-CIT complex (which is calculated by the Hyperquad2006 software); *K* denotes the binding constant; while [*A*]_0_ and [*C*]_0_ denote the total concentrations of albumin and CIT, respectively.

In order to investigate the potential displacement of warfarin (WAR) and ochratoxin A (OTA) from HSA in the presence of citrinin, our previously published models were applied with slight modifications: (1) increasing CIT concentrations (0–5 μM) were added to standard amounts of WAR (1 μM) and HSA (3.5 μM) in PBS (pH 7.4), then the fluorescence emission spectra were recorded (λ_exc_ = 317 nm) [20]; and (2) Increasing CIT or WAR concentrations (0–30 μM) were added to standard amounts of OTA (1 μM) and HSA (1.4 μM) in PBS (pH 7.4), then fluorescence polarization values of the samples were measured (λ_exc_ = 393 nm, λ_em_ = 446 nm) [25,26].

### 3.3. Thermodynamic Studies

To get deeper insight into the formation of CIT-HSA complex, quenching experiments were also performed at different temperatures (25, 30, 35, and 40 °C). Thermodynamic parameters were quantified applying the van’t Hoff equation:

(2) $$\ln K=-\frac{\Delta G}{RT}=-\frac{\Delta H}{RT}+\frac{\Delta S}{R}$$

where *ΔG* is the Gibbs free energy change, *ΔS* is the entropy change, while *ΔH* is the enthalpy change associated with the complex formation.

### 3.4. Ultrafiltration Studies

Before ultrafiltration samples contained 1 μM CIT with or without HSA (1, 2.5 or 5 μM), while displacement of CIT by WAR (5 and 10 μM) or OTA (2.5 and 5 μM) was investigated in the presence of 1 μM CIT and 5 μM HSA. During ultrafiltration experiments Amicon Ultra-4 centrifugal filter units (with 10 kDa molecular weight cut-off value; from Merck Millipore, Darmstadt, Germany) were applied. In order to remove glycerol from the filters, filter units were washed with 3 mL water and 3 mL PBS. Thereafter, 2.5 mL of the samples were transferred into the filter and Amicon tubes were centrifuged at 7500 g for 10 min at 25 °C (in Hettich Universal 32R centrifuge with fixed-angle rotor). Then 2 mL quantity of filtrate was transferred into 5-mL Eppendorf tube, after which 10 μL 6 M HCl solution was added to the sample in order to adjust the pH (to approximately 2). This step is necessary because CIT shows strong fluorescence properties only at highly acidic conditions [19]. Final CIT concentrations were determined (applying calibration curve at concentration range 0.1–2.0 μM) by Hitachi F-4500 fluorescence spectrophotometer with the measurement of the fluorescence signal of CIT itself, using 330 nm and 500 nm as excitation and emission wavelengths, respectively. No spectral interferences were observed during these experiments (data not shown). Regardless of the concentration used (tested between 0.1–2.0 μM CIT concentrations), constant recovery of CIT was determined after ultrafiltration (61.7% ± 1.8%).

### 3.5. Molecular Modeling Studies

Blind docking [32,33,34] calculations were performed using the AutoDock 4.2 [35] program package. Ligand molecules were built in Maestro [36]. It is known that two tautomeric form of citrinin exists in solution (p-quinone and o-quinone; Figure 5D) at room temperature [19,37], therefore both forms were studied in docking calculations. Both tautomeric forms were used in the carboxyl deprotonated state. Energy-minimization of the ligand molecules was performed by the semi-empirical quantum chemistry program package, MOPAC [38], performing a geometry optimization with a 0.001 gradient norm and subsequent force calculations with PM6 parameterization. In all cases, the force constant matrices were positive definite. The apo structure of human serum albumin (pdb code 1a06, Figure 5A) was used as a target of blind docking. Gasteiger-Marsilli partial charges were added to both ligand and target atoms and a united atom representation was applied for groups with non-polar bonds. ALamarckian genetic algorithm was used for search [32]. Docking box was centered on the center of mass of the target. A grid map with a box size of 250 × 250 × 250 points and 0.375 Å spacing was calculated by AutoGrid 4 [35]. The number of docking runs was set to 100, numbers of energy evaluations and generations were 20 million [34]. Ligand conformations resulted from the docking runs were ordered by the corresponding calculated *ΔG* values and clustered [34] using a tolerance of 1.75 Å root mean square deviation (RMSD) between cluster members. Conformations with the lowest binding energy within a cluster were selected as cluster representatives. In this way, blind docking resulted in a list of representative ligand positions and the corresponding calculated *ΔG* values. Ligand-protein contacts were listed and analyzed, within a 3.5 Å distance cut-off between heavy atoms of docked ligands and the target.

### 3.6. Tissue Culture and Cell Viability Measurements

MDCK (immortalized, epithelial-kidney, ATCC: CCL 34) cells were cultured in DMEM containing FBS (10%), penicillin (100 U/mL) and streptomycin (100 μg/mL), in 25 cm^2^ sterile plastic flasks (VWR) and in 24-well sterile plastic plates (VWR). In order to test the effect of CIT on cell viability in absence and presence of albumin, cell media with and without 10% FBS or 40 g/L HSA were also prepared. After 24-h treatment of MDCK cells (in 24-well plates) with CIT (0–100 μM) in absence and presence of FBS (10%) or HSA (40 g/L), cells were washed three times with 500 μL PBS. Then PBS was replaced with 500 µL Hanks + glucose buffer containing 1.6 µM calcein acetoxymethyl ester and cells were incubated for 40 min at 37 °C in the dark. Thereafter, wells were washed again with 500 μL ice-cold PBS and the emptied wells were filled with 500 μL of 5% perchloric acid (PCA) after which cells were incubated for 15 min at room temperature. 20 μL PCA extract was added to 180 μL of 1 M NaOH using black 96-well optical plates (VWR) for fluorescence measurement of the formed calcein (λ_exc_ = 490 nm, λ_em_ = 520 nm). Fluorescence intensities were measured with a microplate reader (Perkin Elmer EnSpire Multimode reader).

### 3.7. Statistics

Statistical analyses were performed by One-Way ANOVA test employing IBM SPSS Statistics 22.0 software. The level of significance was set as *p* < 0.05.

## 4. Conclusions

In summary, in our study the complex formation of citrinin with human serum albumin was investigated. The strong quenching of the fluorescence of HSA by CIT as well as the spectroscopic and ultrafiltration experiments with site markers (warfarin and ochratoxin A) strongly suggest that the high-affinity binding site of CIT is located on Sudlow’s site I (subdomain IIA). This experimental result was also confirmed by molecular modeling studies. Thermodynamic and modeling studies suggest that H-bonds, salt bridges, and hydrophobic interactions are involved as binding forces. Furthermore, based on our results CIT-HSA interaction is an enthalpy-driven process; therefore, electrostatic forces play a major role during the CIT-HSA complex formation. CIT forms a stable complex with HSA (log*K* ~ 5.3); cell experiments indicated the high biological importance of the interaction as well. There are some differences between the complex stabilities of CIT complexes with albumins of other species (bovine, porcine, and rat albumins were tested); however, we did not observe dramatic differences, like in the case of ochratoxin A (which possesses a similar chemical structure to CIT).

## Supplementary Materials

The following are available online at www.mdpi.com/2072-6651/7/12/4871/s1.
