# Supplementary material for: Interaction of Citrinin with Human Serum Albumin
Source: Toxins (Basel). 2015 Dec 1;7(12):5155–66. doi: 10.3390/toxins7124871 (PMC4690121; doi:10.3390/toxins7124871)
Supplement: Supplementary file 1 [file toxins-07-04871-s001.pdf]

Supplementary Materials: Interaction of Citrinin with Human Serum Albumin

Miklós Poór, Beáta Lemli, Mónika Bálint, Csaba Hetényi, Nikolett Sali, Tamás Kőszegi and Sándor Kunsági-Máté

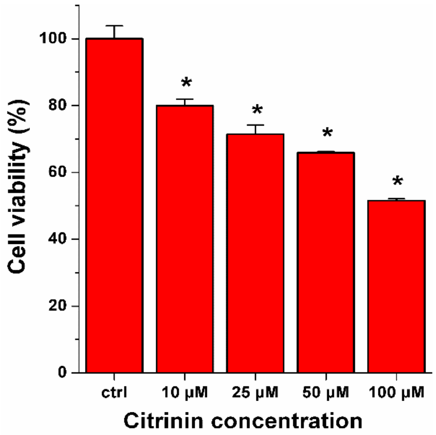

Figure S1. Effects of increasing CIT concentrations on viability of MDCK cells after 24-h treatment in FBS-free medium (* $p < 0.05$).
